# Sulphur K*β* emission spectra reveal protonation states of aqueous sulfuric acid

**DOI:** 10.1038/srep21012

**Published:** 2016-02-18

**Authors:** Johannes Niskanen, Christoph J. Sahle, Kari O. Ruotsalainen, Harald Müller, Matjaž Kavčič, Matjaž Žitnik, Klemen Bučar, Marko Petric, Mikko Hakala, Simo Huotari

**Affiliations:** 1 University of Helsinki, Department of Physics, Helsinki, FI-00014, Finland; 2 European Synchrotron Radiation Facility, ESRF, Grenoble, France; 3 Jožef Stefan Institute, Jamova cesta 39, SI-1001 Ljubljana, Slovenia; 4 Faculty of Mathematics and Physics, University of Ljubljana, Jadranska ulica 19, Ljubljana, Slovenia

## Abstract

In this paper we report an X-ray emission study of bulk aqueous sulfuric acid. Throughout the range of molarities from 1 M to 18 M the sulfur K*β* emission spectra from H_2_SO_4_ (aq) depend on the molar fractions and related deprotonation of H_2_SO_4_. We compare the experimental results with results from emission spectrum calculations based on atomic structures of single molecules and structures from *ab initio* molecular dynamics simulations. We show that the S K*β* emission spectrum is a sensitive probe of the protonation state of the acid molecules. Using non-negative matrix factorization we are able to extract the fractions of different protonation states in the spectra, and the results are in good agreement with the simulation for the higher part of the concentration range.

Sulfuric acid is crucial in the formation of new particles in the atmosphere^1^,^2^. According to the international panel for climate change report, IPCC 2013^3^, the net effect of aerosols is cooling, which compensates for the greenhouse effect caused by rising levels of antropogenic CO_2_ in the atmosphere. The bulk and cluster systems of aqueous sulfuric acid have attracted considerable theoretical (for example, refs 4, 5, 6, 7, 8, 9, 10, 11, 12, 13, 14, 15, 16, 17, 18, 19, 20, 21, 22, 23, 24, 25, 26, 27, 28, 29, 30) and experimental (for example, refs 30, 31, 32, 33, 34, 35, 36, 37, 38, 39) interest. One frequently appearing question in these works considers the dynamics of the protons, which may be very relevant from the point of view of atmospheric new particle formation^40^,^41^. Apart from atmospheric importance, sulfuric acid is a widely used chemical in industry. Aqueous sulfuric acid is a dynamic system, that undergoes chemical reactions in its equilibrium; due to its ability to donate protons, covalent chemical bonds are formed and broken continuously.

X-ray spectroscopies are element and site specific probes to obtain atomistic information on molecular liquids and clusters. The core-excitation (absorption) and decay (emission) spectra are indeed sensitive to particular elements of choice and their immediate chemical environment owing to orbital energetics. The emission spectrum provides spectral fingerprints of the chemical environment and the initial K shell core hole can be created by photon or electron impact. In essence, changes in the occupied valence orbitals, chemically binding the molecule, are seen as changes in the decay spectra. This effect can be used as a fingerprint of atomistic structure^42^,^43^. Molecular liquids are an interesting class of samples for core-excitation and emission studies and, for example, X-ray photoelectron spectroscopic work on sulfuric acid was recently reported^39^ in addition to works on sulphate compounds in the condensed phase other than H_2_SO_4_ 44,45. We have also recently studied aqueous H_2_SO_4_ using first-principles simulations and core-level excitations by inelastic X-ray scattering^30^.

In this work, we report on our study of the S K*β* emission spectra of aqueous sulfuric acid as a function of the H_2_SO_4_ concentration. With the help of calculations we show that the protonation state of the acid is manifested in the emission spectrum, mimicking the local orbital structure of the molecular valence. Our results show that the S K*β*_*x*_ emission line originates from acid molecules with one or two protons and that the spectral shape is a sensitive probe of the protonation state of the acid molecules. We use the non-negative matrix factorization method (NNMF)^46^,^47^ to quantify the percentages of different protonation states in the system, and the results are in agreement with *ab initio* molecular dynamics structural simulations for the higher part of the concentration range.

## Results and Discussion

**Figure 1:**
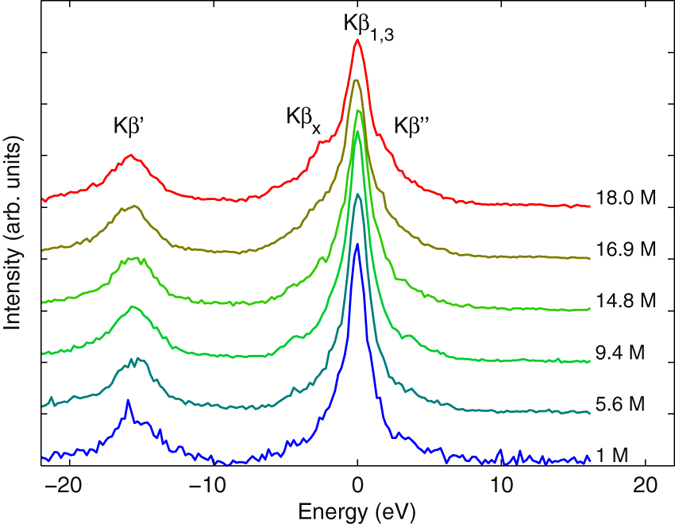
The S K*β* emission spectra of aqueous sulfuric acid as a function of concentration. A relative energy scale is used with the origin at the K*β*_1,3_ line (2465 eV).

Figure 1 presents the experimental emission spectra of aqueous sulfuric acid. Alonso-Mori and coworkers studied the S K*β* emission spectrum of sulphate minerals and assigned the features in the spectrum^45^ based on orbital structure calculations. We follow the usual nomenclature in our discussion and assign lines in the spectra of Fig. 1 as K*β*’ and K*β*_1,3_. The latter line has shoulder-like structures K*β*_*x*_ and K*β*”. Moreover, as seen clearly in the 18.0 M system, the K*β*_*x*_ and K*β*” features both have two components. With increasing concentration, the highest K*β*_1,3_ line loses intensity, and the K*β*_*x*_ and K*β*” lines gain spectral weight. We assign this behavior to two phenomena: the protonation state of the acid and the effect of solvation.

**Figure 2:**
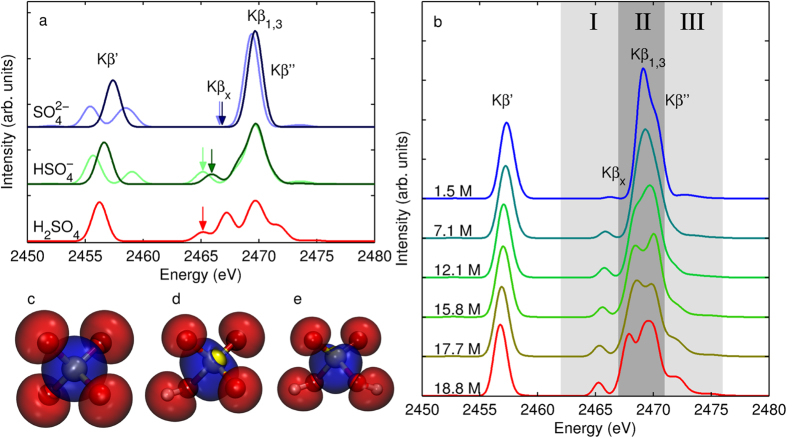
(**a**) Calculated S K*β* emission spectra of 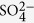, 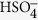, and H_2_SO_4_ in gas phase. The lighter color represents the spectra of 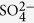, 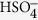 without re-optimization of the geometry, and the darker colour represents the spectra with geometry optimization for the particular species. The arrows show the position of the line originating from orbital 14. (**b**) Calculated S K*β* emission spectra of aqueous sulphuric acid in the range from 1.5 M to 18.8 M, based on *ab initio* molecular dynamics simulations. The calculated orbital 14 (in the ground state) responsible for lowest transition of the K*β*_*x*_ line in the emission spectra of (**c**) 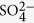, (**d**) 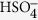, and (**e**) H_2_SO_4_.

To gain a deeper understanding of the effects of the protonation state on the shape of the spectrum, we performed spectrum calculations for single molecules in vacuum, presented in Fig. 2(a), and calculations based on *ab initio* molecular dynamics (AIMD) simulations, presented in Fig. 2(b). For the in-vacuum single-molecule calculations the used structures are presented in Fig. 2(c–e). The calculation was performed for a geometry optimized H_2_SO_4_ molecule and systems obtained by removal of one or two protons from the molecule and reoptimizing the geometry. Moreover we performed calculations removing the proton(s) but without re-optimization of geometry for 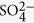 and 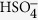. The results of the latter calculations are shown in light color in Fig. 2(a). The number of electrons and occupied orbitals remained constant in all three systems. The orbitals are sensitive to protonation, which is manifested in the individual transitions in the emission spectrum. For H_2_SO_4_, the K*β*’ line consists of 3 transitions, which are strongly split in the deprotonated but non-reoptimized systems, and which are regrouped after geometry re-optimization. The K*β*_*x*_ − K*β*_1,3_ − K*β*” group has 5 transitions, and the spectral shape of this feature is not largely affected by the geometry re-optimization. Moreover, the splitting of the latter lines occurs in H_2_SO_4_, both the K*β*_*x*_ and the K*β*” features being reproduced by the calculations.

The in-vacuum calculation reveals two-fold effects, when protons are removed from the acid. First, orbital eigenenergies and even their order in energy may be modified, and second, intensity changes due to symmetry and due to reshaping of the orbitals can occur. The latter is manifested in Fig. 2(a) for the lowest part of the K*β*_*x*_ line. In the 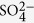 ion (Fig. 2(c)), the orbital from which the decay takes place is spherical around the S atom, making the transition to S1s dipole forbidden. As one or two protons are added (Fig. 2(d,e)), breaking of the symmetry causes the orbital to change shape, which makes it allowed for decay by dipole transition. This analysis applies strictly only to the high-symmetry geometry of 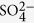, and, due to ensemble averaging, the transition becomes also allowed in real systems, yet probably very weakly.

Secondly, as a more precise but also more challenging approach, we simulated ensemble-averaged S K*β* emission spectra by sampling the trajectories from previously published AIMD simulations^30^. The protonation distribution along the AIMD runs is presented in Table 1. The obtained AIMD-based vertical emission spectra are depicted in Fig. 2(b). The simulation shows again a single peak for the K*β*’ line. Moreover, the simulation for the 1.5 M system reproduces the K*β*” satellite. For the 18.8 M system, the K*β*_*x*_ − K*β*_1,3_ − K*β*” line group resembles the in-vacuum H_2_SO_4_ case owing to the fact that the system consists mostly of the fully protonated H_2_SO_4_. The 18.8 M case produces the further splittings of lines seen in Fig. 2(b). The origin of this is apparent from the spectrum calculation of the isolated H_2_SO_4_ molecule in vacuum as shown in Fig. 2(a). The simulation of Fig. 2(b) shows qualitative agreement with the experiment, with some discrepancy in reproduction of the K*β*_*x*_ and K*β*”, lines. The origin of the mismatch is most likely due to spectrum simulation. For the 1.5 M case, in turn, the structural simulation gives erroneous results, as discussed later in our NNMF analysis.

**Figure 3:**
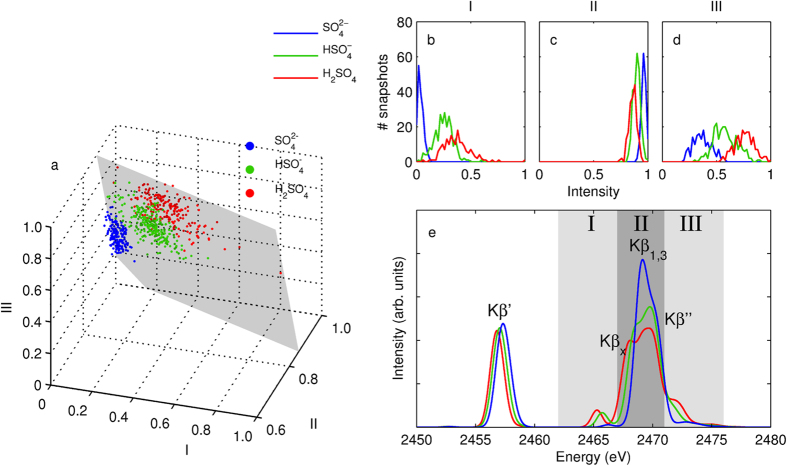
(**a**) The individual-snapshot spectra presented in a plot of three intensity values I–III for all snapshots of all concentrations. Coloring refers to protonation state of the excited acid molecule and the grey plane presents the best fit using a plane. The values have been normalized to 1 corresponding the maximum. The intensity distributions in regions (**b**) I, (**c**) II, and (**d**) III, with respect to protonation state of the acid molecule. Again, the values are normalized to the maximum intensity of any snapshot in the region. (**e**) The averaged spectra of the three different forms of acid based on the 600 snapshot total.

To study the statistical process of formation of the ensemble-averaged spectrum from the spectra of individual configurations, we analyzed the intensities in regions I-III of Fig. 2(b) for each snapshot in the liquid simulation. By integrating the intensity in each of the regions, that were chosen from an averaged spectrum, we transform the complex problem of relating spectral features and changes thereof to structural properties into a more feasible one. This allows studying spectral structure – property relationships, naturally depending on how much correlation between the two exists. The data points of all the concentrations are depicted maximum-normalized in Fig. 3(a), and they lie close to a plane in I,II,III intensity-space. The grouping of the points follows the protonation state of the acid molecule, which is known from the simulation for each data point.

The distribution of intensity values in the different regions I-III for the three protonation forms is presented in Fig. 3(b–d) for all concentrations at once. The figure shows progression in all of the three regions as a function of the protonation state, and the intensity values of 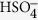 are between those of 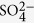 and H_2_SO_4_. In region I (the low K*β*_*x*_) 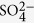 intensity is close to zero for most snapshots and in both region I and III the intensity grows with the number of protons in the acid molecule. For region II, that includes the K*β*_1,3_ peak, the intensity decreases with increasing number of protons.

The number of protons in the emitting acid molecule is known from the simulation (see the Methods section). We calculated the averaged spectra for all three forms, from all snapshots of all concentrations, presented in Fig. 3(e). The analysis reveals that in the bulk case the K*β*_*x*_ and K*β*” features appear in both 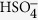 and H_2_SO_4_, but as shown in the histograms of Fig. 3(b–d) the intensities vary along the number of protons. This is in agreement to the in-vacuum case, with the notion that the K*β*” feature appears as a separated peak in H_2_SO_4_ and as a tail of the main line in 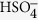.

**Figure 4:**
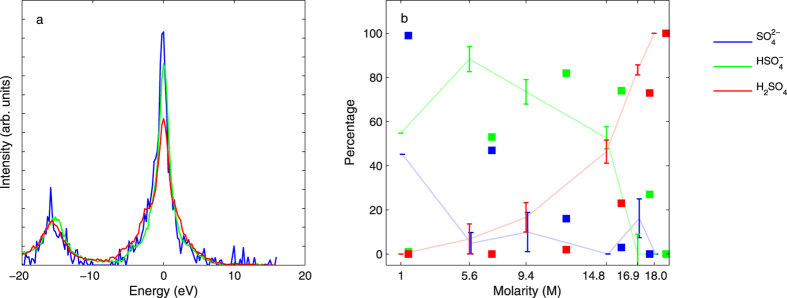
(**a**) The NNMF spectra for 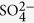, 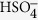, and H_2_SO_4_. (**b**) The weights of the spectra (fractions of molecular species) from NNMF are shown using errorbars and the values from AIMD simulations are show using squares.

We extracted the component spectra of 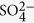, 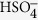, and H_2_SO_4_ and their weights (and therefore molecular percentages) from the experimental spectra using constrained NNMF, see ref. 46 and Methods section for details. The number of components is less than the number of raw spectra, which enables an approximative low-rank matrix factorization of the data set. As constraints we used the assumptions that 18.0 M aqueous sulfuric acid is 100 mol-% H_2_SO_4_ and that the 1 M acid has (45.2–54.8) mixture of 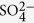 and 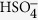, based on the value 1.21 for 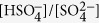 by Margarella and coworkers^39^. The 2 unconstrained spectra are allowed to take any functional form in the procedure, and are “discovered” by the NNMF algorithm, guided only by best describing the data set of 6 spectra using only 3 (one of which is fixed) with varying weights. The NNMF procedure does not provide unique solution to the spectral composition problem. The results depend on the initial guesses used, which in the current case were the 1 M and the 9.4 M spectra for 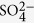 and for 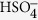, and the 18.0 M spectrum (fixed) for H_2_SO_4_. Apart from the fixed coefficients, the initial guesses of the coefficients were taken from the simulations. The shapes of the resulting component spectra however suggest that the result is reasonable. The NNMF-obtained component spectra in Fig. 4(a) manifest similar behaviour in terms of the ratios K*β*_*x*_/K*β*_1,3_ and K*β*_*x*_/K*β*″ as the simulation, which supports the interpretation. Figure 4(b) presents the coefficients of the different spectra with the values from AIMD simulations, and for the higher concentrations the agreement is found to be good. The most likely reason for the mismatch at lower concentration is the quality of the structural AIMD simulation, as we observe large discrepancies between different computational works^14^,^30^, both of which disagree with the experimental value^39^. We conclude that the lowest concentrations are difficult to simulate due to most likely long reaction times for the span of AIMD. The NNMF coefficients are not in complete agreement with previous experiments by Margarella and coworkers^39^. A possible reason for this may be the used NNMF procedure, or its inherent assumption that there are three component spectra.

In conclusion, the S K*β* emission spectra of aqueous sulfuric acid changes with the protonation state of the acid molecule. This is due to the changes in the valence orbitals, that also chemically bind the protons. The intensities of the K*β*_*x*_, K*β*_1,3_, and K*β*″ are a probe of the protonation state of the acid, and the lowest K*β*_*x*_ emission line originates from acid molecules with one or two protons. The obtained molecular fractions from the experiment are in agreement with those from AIMD simulation, apart from the lowest studied concentrations. The overall trends in the spectral changes from protonation state to other are explained by the spectrum calculations.

## Methods

The experiment was performed at the ID26 beamline of the European Synchrotron Radiation Facility (ESRF). The incident photon energy was tuned by means of a cryogenically cooled double Si (111) crystal monochromator with the resolution of 0.36 eV. Higher harmonics were suppressed by two Si mirrors operating in total reflection. We utilized a closed acid-resistant sample cell to confine liquid sulfuric acid inside an emission spectrometer operating in vacuum conditions. The photon beam with a 50 × 250 *μ*m^2^ cross section was directed on a closed acid-resistant PTFE sample cell with 1.0 *μ*m thick 1 × 1 mm^2^ Si_3_N_4_ front window, which allowed for conditions very close to room/ambient conditions inside the cell. X-ray fluorescence was collected along the polarization direction of the incident photon beam and analyzed with a Johansson type in-vacuum x-ray emission spectrometer^48^. The spectrometer operated in the dispersive geometry that combines target positioning within the Rowland circle with a position sensitive detection of diffracted x-rays. In our experiment, the target cell was placed at a distance of 42 cm in front of the diffraction crystal. The first order reflection of the Si (111) crystal was used, and the diffracted photons were detected by a thermoelectrically cooled (−40 °C) CCD camera consisting 770 × 1152 pixels with 22.5 × 22.5 *μ*m^2^ pixel size. The experimental resolution at the energy of the S K*β* emission line was 0.45 eV and the bandwidth collected at the fixed detector position was ≈40 eV which enabled us to collect whole K*β* emission spectrum simultaneously.

Sulphuric acid with 18.0 M was provided by Carlo Erba Reagents and solutions with lower concentrations were purchased from Carl Roth. We used commercially provided solutions without further processing, except for the 14.8 M (79-m%) solution, which we prepared by using 3 g of 11.6 M (62-m%) and 3 g of 18.0 M (96-m%) solutions. The obtained spectra were brought onto a releative energy scale centered at the K*β*_1,3_ peak.

To gain physical insight to the molecular structures, we evaluated X-ray emission spectra for the studied systems. The emission energies and intensities were obtained by two separate calculations: (i) the single molecules H_2_SO_4_, 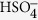, and 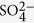 in vacuum, (ii) ensemble-averaged spectra of the bulk liquids using snapshots from restricted Kohn-Sham (RKS) *ab initio* molecular dynamics (AIMD) simulations reported earlier^30^. The number of atoms in the box was approximately 200, depending on the concentration.

For in-vacuum calculation, we utilized the augmented correlation-consistent polarized valence quadrupole-*ξ* (aug-cc-pVQZ) basis set by Dunning and coworkers^49^,^50^ for all atoms. The structures were obtained by geometry optimization for H_2_SO_4_ and by removal of one or two protons. For 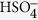 and 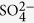 two calculations were made. The first one models the effect of removing proton(s) from H_2_SO_4_ without additional geometry optimization, and the second one contains also affects from geometric relaxation. For generation of orbital plots, the code package ERKALE^51^,^52^ was used. The orbital was taken from ground-state calculation and the orbital ordering number of this orbital was assumed to remain the same in XES, which is supported by an orbital calculation using the Z + 1 approximation. The ground-state eigenenergies for orbitals in CP2K^53^ and Erkale match well. The orbital plots were drawn using the VMD package^54^

All spectral calculations, except calculations for the orbital plots, were done using the CP2K code package^53^. The calculations use the Gaussian and the augmented plane wave method of ref. 55. The AIMD simulations were performed in our previous work^30^ for 6 different concentrations that we modelled by combinations of (1 A, 63 W), (6 A, 54 W), (12 A, 36 W), (20 A, 20 W), (21 A, 7 W) and (24 A, 0 W) acid (A) - water (W) molecules in the simulation box. The choice corresponds to the molarities 1.5 M, 7.1 M, 12.1 M, 15.8 M, 17.7 M, and 18.8 M and for spectrum calculations in this work, 100 structures for each concentration were taken from the trajectories. For the protonation analysis, averages over the production run trajectories were calculated for each of the simulated concentration. A proton was considered to be attached to the 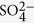 if its distance to any of the oxygens was less than 1.3 Å, a criterion also used by Choe *et al.*^14^. An oxygen atom in turn, was determined to belong to the 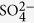 if its distance to the sulphur atom was less than 2.0 Å. The delta-peak spectra from the calculation were finally convoluted with a Gaussian line shape with a full width at half maximum of 1.5 eV. For the set (i), this FWHM was used to account for statistical broadening expected in the system, incoming photon bandwidth, spectrometer resolution, and life-time broadening. For the set (ii) the statistical broadening is covered by the phase space sampling and we used a FWHM of 1.0 eV instead. For all bulk spectrum simulations, we used the aug-cc-pVTZ basis set for the excited S atom and the TZVP-MOLOPT-GTH basis set^56^ with GTH pseudopotentials^57^ for the other atoms. The Perdew-Burke-Ernzerhof (PBE) exchange-correlation functional^58^ was used in all calculations.

We performed constrained non-negative matrix factorization (CNNMF) of the experimental spectra to extract percentages of the different molecular species. Non-negative matrix factorization (NNMF)^46^,^47^ is a method that can be used to represent a series of *m* spectra of *n* energy points with *k* < *m* non-negative components and their non-negative weights. Formally one obtains a matrix factorization


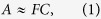


where *A*_*n*×*m*_, *F*_*n*×*k*_, and *C*_*k*×*m*_ are the non-negative matrices containing the raw data, the component spectra, and their weights, respectively. The factorization is not exact and all spectra are integral-normalized to unity. To achieve optimization with constraints, we made our own implementation of the steepest descent (SD) algorithm. We minimize the quadratic cost function


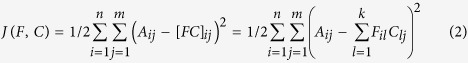


with respect to matrix elements of *F* and *C*. The gradient of (2) then becomes


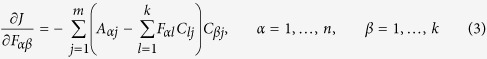


and


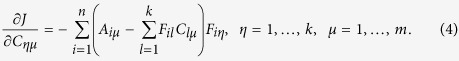


Here the cost function was optimized using SD algorithm


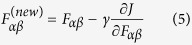


and


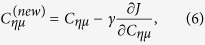


where *γ* = 0.01 scaling parameter was used. Finally in the end of the iteration cycle, all negative elements were set to 0, a procedure listed as one alternative by Berry and co-workers^46^. Constrained optimization was achieved by giving the values of constrainted elements as initial guesses and never updating these elements in the SD iterations. Thus the partial derivatives (3)-(4) calculated for elements, were used only for the desired (*αβ*), (*ημ*) pairs in SD. Constraints are desired in NNMF as the matrices *F* and *C* are not unique. The procedure was constrained, by fixing one of the three component spectra to that of 18.0 M H_2_SO_4_ with weight 1 at 18.0 M, and by fixing the coefficients of 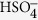 and 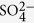 to be 0.548 and 0.452 for 1 M solution, as given by Margarella *et al.*^39^. The statistical errors for the coefficients *C* were obtained using the bootstrap algorithm with 100 resamplings. In the procedure random noise, the magnitude of which is extracted from the experimental spectra (as mean difference of intensity in subsequent energy channels), is added to spectra of *A* and CNNMF is run to obtain new *F* and *C*. The errorbars represent the mean of absolute deviation of the resampled set from the original coefficients *C*.

## Additional Information

**How to cite this article**: Niskanen, J. *et al.* Sulphur Kβ emission spectra reveal protonation states of aqueous sulfuric acid. *Sci. Rep.* **6**, 21012; doi: 10.1038/srep21012 (2016).

## Figures and Tables

**Table 1:** Fractions of the protonation states of sulfate ions calculated as averages from the AIMD production runs by Niskanen *et al.* 30.

Concen.	H_2_SO_4_	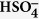	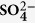
M (mol-%)	mol-%	mol-%	mol-%
1.5 (2)	0 (0.0)	1 (100)	99 (0.0)
7.1 (10)	0	53	47
12.1 (25)	2 (0.0)	82 (89.0)	16 (11.0)
15.8 (50)	23	74	3
17.7 (75)	73	27	0
18.8 (100)	100	0	0

The values by Choe and coworkers^14^ are given in parenthesis.
